# Socioeconomic stratification in adolescent digital engagement: cultural capital, emotional mediation, and bilibili usage patterns in Chinese high schools

**DOI:** 10.3389/fsoc.2025.1696513

**Published:** 2026-01-13

**Authors:** Qiaoyi Liu

**Affiliations:** The School of Economics and Management, Shanghai Institute of Technology, Shanghai, China

**Keywords:** affect-behavior-cognition model, Bilibili, emotional mediation, high school students, structural equation modeling, user behavior

## Abstract

This sociological study examines how cultural capital and institutional structures shape digital behaviors among 606 Chinese high school students using stratified sampling across school types (provincial/municipal/regular) and income groups. Applying Bourdieu's capital theory within an Affect-Behavior-Cognition framework, we reveal entrenched stratification: students from high-income households (≥310,000 CNY) demonstrate significantly stronger cultural identity (Δ*M* = 0.15, *F* = 2.533, *p* = 0.048) and social interaction (Δ*M* = 0.24, *F* = 3.767, *p* = 0.005) compared to low-income peers, while municipal key school students exhibit 12% higher social interaction engagement than regular school counterparts (*F* = 2.694, *p* = 0.031). Parental occupation further mediates cultural capital conversion, with executives' children showing higher cultural identity (Δ*M* = 0.23 vs. service workers, *p* = 0.018). Crucially, emotion (β = 0.939, *p* < 0.001) serves as the mechanism that translates cognitive resources (entertainment β = 0.210, resources β = 0.210) into behavioral capital, yet this mediation pathway is disproportionately accessible to economically advantaged youth. Residential location showed no significant effects, indicating Bilibili's uniform penetration but stratified usage patterns. These findings demonstrate how educational systems and familial capital jointly reproduce digital inequalities, with emotion serving as an overlooked conduit for converting cognitive advantages into behavioral capital. The study advances Bourdieusian theory in platform societies and proposes interventions for democratizing digital habitus through equitable content algorithms and school-based digital literacy programs.

## Introduction

As a leading video-sharing community in China, Bilibili (commonly known in China as B站, pronounced *B-Zhan*) has rapidly become an important digital environment through which high school students engage in learning, entertainment, and identity construction (Yang, 2018; Peng, 2023). Its diverse content ecosystem, strong interactivity, and distinctive subcultural atmosphere have strengthened its appeal to adolescents. However, amid intense competition from short-video platforms such as TikTok (*Douyin*), attention has increasingly been diverted, and user stickiness on Bilibili has declined. Understanding the mechanisms underlying its attractiveness to adolescents has therefore become an urgent, practical and theoretical task. Prior studies suggest that fulfilling multiple usage motivations is essential for sustaining user engagement (Kaveladze et al., 2022), and that, for high school students, platform use reflects a complex interplay of social identification, emotional experience, and interest development (Ilkic et al., 2023). Clarifying the psychological drivers of adolescents' use of Bilibili is thus of considerable value.

Although research on online video platforms has expanded in recent years, covering entertainment motives, learning needs, and technology acceptance (Truong and Van der Wal, 2024), empirical studies focusing specifically on high school students remain limited. The existing literature primarily targets university students or young adults and has yet to systematically investigate the distinctive psychological and behavioral mechanisms observed in adolescents. The role of emotion has been particularly under examined: existing evidence indicates that emotional experience may influence immersion and continued usage (Cheng et al., 2023), yet robust validation in real adolescent contexts is lacking. As Bilibili has evolved from an ACGN-centered community into a comprehensive video platform, user motivations have diversified, and single-variable explanations are no longer sufficient to capture the complexity of user behavior. Filling this research gap requires a systematic examination of the emotional mechanisms shaping high school students' engagement with the platform.

Despite the growing body of work on short-video and social-media usage, Scopus-indexed studies on Bilibili still predominantly focus on content ecology, subcultural communities, and bullet-chat interaction at the macro level (Chen et al., 2022), with limited attention to individuals' psychological and behavioral mechanisms (Zheng et al., 2023; Liu et al., 2024). Existing research highlights identity, community interaction, and learning scenarios but seldom employs structured psychological models to clarify the relationships among cognition, affect, and behavior. Cognitive drivers remain under-theorized, and the synergistic interplay among entertainment, learning, cultural identity, social interaction, and perceived convenience has not been adequately addressed (Tu et al., 2025; Wang et al., 2025). Meanwhile, international studies on TikTok and YouTube have linked short-video use with adolescent mental health and emotional experience (Conte et al., 2024; Turuba et al., 2024), yet these studies remain largely descriptive and rarely test the mediating role of emotion in the cognitive-behavioral process. Against the backdrop of intensifying competition and the continuous fragmentation of adolescent attention, it is essential to examine adolescents' use of Bilibili through an integrated Affect-Behavior-Cognition (ABC) framework.

The ABC model has been widely applied in consumer psychology and user-behavior studies (Yingqing et al., 2024). Within this framework, cognitive variables such as learning, entertainment, and social interaction function as antecedents of affective responses, and affect serves as a central mechanism shaping behavioral tendencies. Previous research demonstrates that in high-involvement or high-engagement contexts, the affective pathway mediates the relationship between cognition and behavioral intention, and may even exert stronger predictive power than cognitive evaluations themselves (Keer et al., 2010; Mohamad et al., 2019). However, existing evidence is mainly derived from adult or university samples, limiting its generalizability to adolescents.

To address these gaps, this study investigates whether six cognitive dimensions, learning, entertainment, social interaction, cultural identity, resources, and perceived convenience, affect high school students' behavioral tendencies toward Bilibili indirectly through affect, thereby clarifying the mediating role of emotion in the cognition-behavior relationship. Using a large-scale survey of high school students across diverse regions in China, we construct a comprehensive path model to identify how multidimensional cognitive factors and affective experiences jointly shape platform usage behavior. Theoretically, this study is the first to systematically incorporate the ABC model into the Bilibili–adolescent context, establishing a structured ABC framework that addresses a major gap in digital-youth research and extends the ABC model's applicability. In practice, our findings provide evidence-based insights for optimizing the adolescent user experience and enhancing user retention amid escalating competition in short videos. They also offer implications for content curation, social interaction design, and cultural-identity strategies across other digital platforms targeting youth. At the societal level, this study sheds light on the psychological mechanisms underlying adolescents‘ digital engagement and contributes to fostering a healthier online cultural ecosystem.

## Methods

### Research design and procedure

This study employed a cross-sectional survey design (Supplementary material) grounded in the ABC model. It examined how six cognitive dimensions—learning, entertainment, social interaction, cultural identity, resources, and convenience—influence affect, and further assessed the mediating role of affect in the relationship between cognition and behavior.

To ensure systematic rigor and methodological transparency, the research process followed a structured sequence: questionnaire development and pilot testing → sample selection and data collection → data cleaning and quality control → reliability and validity testing → descriptive statistical analysis → correlation and variance analyses → path and mediation analysis → robustness checks. All procedures adhered to established ethical standards in educational research, ensuring the scientific integrity and reproducibility of data collection and analysis.

Before data collection commenced, an overall flowchart was created to illustrate the study's logic and core steps (Supplementary Figure S1). The flowchart provides a clear representation of the process from questionnaire development to model validation, thereby facilitating replication and extension within similar frameworks. This design aligns with international expectations for methodological transparency and logical coherence in high-quality academic research.

### Participants and sampling

The participants were Chinese high school students enrolled during the 2023–2024 academic year. To enhance representativeness, a combination of stratified random sampling and convenience sampling was employed. Stratification was based on geographic region (eastern, central, western, and northeastern China), city tier (first- to fourth-tier), and school type (provincial key, municipal key, district key, and regular high schools). Within each stratum, schools were randomly selected, and students were subsequently invited to participate through convenience sampling (Supplementary Figure S2).

A total of 612 questionnaires were distributed. After excluding incomplete or logically inconsistent responses, 606 valid questionnaires were retained, yielding a valid response rate of 99.0%. As summarized in Table 1, the demographic profile of the sample was as follows: gender−215 male students (35.48%) and 391 female students (64.52%); grade level−255 first-year (42.08%), 112 second-year (18.48%), and 239 third-year students (39.44%). Family income distribution comprised 101 students from low-income households ( ≤ 100,000 CNY), 372 from middle-income households (110,000–300,000 CNY), and 133 from high-income households (≥310,000 CNY). The diversity of regions and school types represented in the sample supports the generalizability of the findings to the wider population of Chinese high school students.

**Table 1:** Demographic characteristics of the study population.

**Characteristic**	**Statistic**	**Frequency**	**Proportion**
Gender	Male	215	35.48%
	Female	391	64.52%
Grade	Sophomore	112	18.48%
	Junior	255	42.08%
	Senior	239	39.44%
Academic performance	Top 5% of the grade	75	12.38%
	6% to 25% of the grade	270	44.55%
	25% to 50% of the grade	192	31.68%
	51% to 75% of the grade	52	8.58%
	Bottom 24%	17	2.81%
Currently enrolled school	A. Provincial key high school	100	16.5%
	B. Municipal key high school	256	42.24%
	C. District key high school	151	24.92%
	D. Ordinary high school	99	16.34%
Highest level of education of either parent	A. High school or less	119	19.64%
	B. College diploma	123	20.3%
	C. Bachelor's degree	282	46.53%
	D. Graduate degree or above	82	13.53%
Occupation of either parent	A. Government official	53	8.75%
	B. Staff at scientific, educational, cultural, or health institutions	132	21.78%
	C. Business owner	53	8.75%
	D. Corporate manager	133	21.95%
	E. Company employee	138	22.77%
	F. Service industry worker	23	3.8%
	G. Freelancer or self-employed	74	12.21%
Annual family income	A. Less than 100,000 RMB	101	16.67%
	B. 110,000 to 200,000 RMB	191	31.52%
	C. 210,000 to 300,000 RMB	181	29.87%
	D. 310,000 to 400,000 RMB	76	12.54%
	E. 410,000 to 500,000 RMB	57	9.41%
Place of residence	A. Beijing, Shanghai, Guangzhou, and Shenzhen	144	23.76%
	B. Other first-tier cities and provincial capitals outside of Beijing, Shanghai, Guangzhou, and Shenzhen	180	29.7%
	C. Second-tier cities	143	23.6%
	D. Third and fourth-tier cities and towns	139	22.94%

This table presents the distribution of 606 high school students by gender, grade level, family income, parental education, parental occupation, and residential location. Data are reported as frequencies (n) and percentages (%). The results indicate broad coverage and balanced distribution, with higher proportions of females, lower-grade students, and middle-income groups.

### Questionnaire design and measurement instruments

The questionnaire was developed based on the ABC model and comprised eight dimensions: learning, entertainment, social interaction, cultural identity, resources, convenience, affect, and behavior (Table 2). Each dimension contained four to six items measured on a five-point Likert scale (1 = strongly disagree, 5 = strongly agree). For example, the learning dimension included the item, “Bilibili content helps me complete learning tasks,” while the affect dimension included the item, “I feel enjoyment and a sense of identification while using the platform” (Supplementary Figure S3).

**Table 2:** Questionnaire design and measurement indicators.

**Variable names**	**Measurement indicators**
Learning	• Compared to other software, I prefer learning on Bilibili. • On Bilibili, compared to other software, tutorial-related videos are recommended to me. • My learning efficiency on Bilibili is higher than on other software.
Entertainment	• Compared to other software, I get more relaxation on Bilibili. • On Bilibili, I can access more entertainment information compared to other software. • I can watch more entertainment programs on Bilibili than on other software.
Community culture	• Compared to other software, I prefer the bullet comments on Bilibili. • Compared to other software, I prefer interacting with friends on Bilibili. • Compared to other software, I prefer submitting content on Bilibili.
Convenience	• Compared to other software, I prefer the unique resources available on Bilibili. • Compared to other software, I prefer watching videos that are shared on Bilibili. • Compared to other software, I prefer watching videos that premiere on Bilibili.
Preferences	• Overall, compared to other software, I believe Bilibili better represents the culture of the new generation of young people. • Overall, compared to other software, I believe Bilibili is good at guiding teenagers toward the right path. • I use Bilibili as frequently as my classmates do. • I am very willing to recommend Bilibili to my classmates. • I will continue to use Bilibili.

A pilot test was conducted with 60 high school students before the formal survey. Item wording was refined based on respondents' feedback and psychometric evaluation, including Cronbach's α and the KMO measure, to ensure clarity and validity. In the formal survey, reliability and validity analyses showed strong internal consistency (overall Cronbach's α = 0.931; subscale α values ranging from 0.81 to 0.90). The KMO value was 0.957, and Bartlett's test of sphericity was significant (χ^2^ = 8,453.21, df = 820, *p* < 0.001), confirming suitability for factor analysis and strong construct validity.

### Data collection and quality control

Data were collected online using the Wenjuanxing platform (https://www.wjx.cn/). To minimize sampling bias, questionnaires were distributed through multiple channels, including teachers, parents, and peers. Participation was voluntary, with informed consent obtained from both respondents and their guardians. Strict anonymity and confidentiality were maintained, and data were used exclusively for research purposes.

To ensure authenticity and reliability, several exclusion criteria were applied: (1) completion time under 120 s; (2) logical inconsistencies or missing values; and (3) identical responses across all items. After cleaning, 606 valid questionnaires were retained. Additionally, a dual independent verification process was conducted before data entry and analysis to further reduce potential errors.

### Variable specification and grouping criteria

In the model, independent variables included learning, entertainment, social interaction, cultural identity, resources, and convenience. Affect served as the mediating variable, while user behavior—operationalized as platform stickiness and activity level—was the dependent variable. Control variables comprised gender, grade, family income, parental occupation, school type, and region, which were also used in subgroup analyses.

Grouping criteria were defined as follows: gender (male, female); grade (first, second, and third year); family income (low, medium, and high); and school type (provincial key, municipal key, district key, and regular high school). This stratification enabled a more nuanced examination of differences in Bilibili usage across diverse demographic and socioeconomic backgrounds.

### Statistical analysis

All analyses were conducted using IBM SPSS v21.0 and AMOS v21.0, with the level of significance set at *p* < 0.05. Descriptive statistics were first employed to summarize the distribution of demographic characteristics (frequencies, percentages) and to calculate the means and standard deviations of each study dimension, providing an overview of high school students' use of Bilibili. Reliability and validity of the measurement instruments were then evaluated. Internal consistency was assessed with Cronbach's α, while the Kaiser-Meyer-Olkin (KMO) measure and Bartlett's test of sphericity were used to confirm the suitability of the data for factor analysis. Confirmatory factor analysis (CFA) was subsequently conducted, with model fit evaluated using χ^2^/df, RMSEA, Comparative Fit Index (CFI), Tucker–Lewis Index (TLI), and Goodness of Fit Index (GFI), ensuring structural stability of the scales.

For inferential statistics, Pearson correlation analysis was applied to examine bivariate associations among learning, entertainment, social interaction, cultural identity, resources, convenience, affect, and behavior. One-way Analysis of Variance (ANOVA) with *post hoc* Bonferroni or Tukey tests was used to identify group differences across gender, grade, school type, and household income. To further assess the impact of socioeconomic factors, hierarchical regression and hierarchical linear modeling (HLM) were performed to test the moderating effects of family income and parental occupation on the affect–behavior pathway. Structural equation modeling (SEM) was then employed to conduct path analysis, testing the influence of six cognitive dimensions on affect as well as the mediating role of affect between cognition and behavior. Model fit criteria were set at χ^2^/df < 3, RMSEA < 0.08, and CFI/TLI/IFI/GFI > 0.90. Mediation effects were verified using the Bootstrap method (5,000 resamples), with 95% confidence intervals reported. Finally, sensitivity analyses—including random exclusion of 10% of cases, regional subgroup analyses, and comparison with alternative models—were conducted to evaluate the robustness and generalizability of the findings.

## Results

### Sample characteristics and distribution

**Figure 1:**
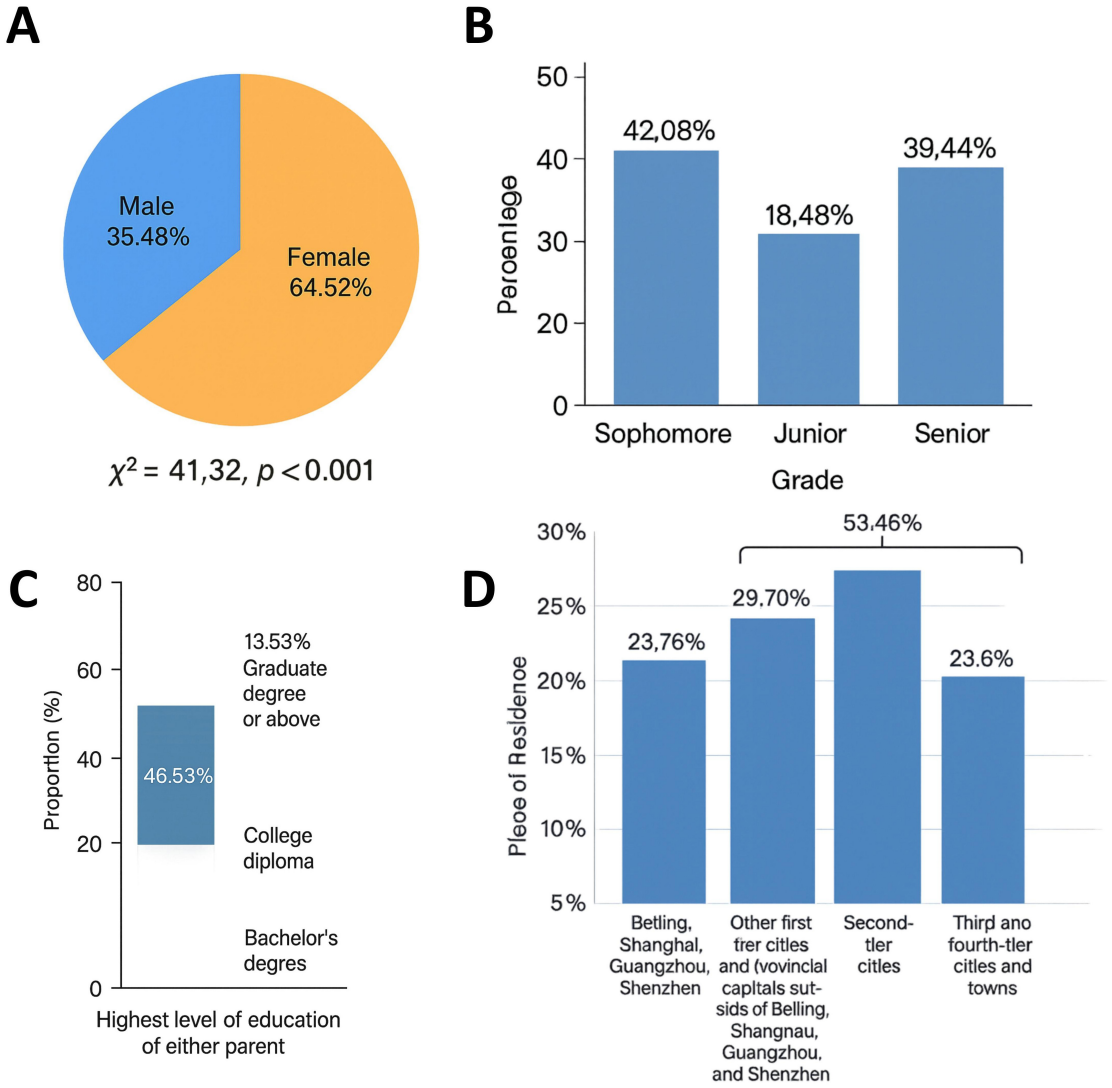
Demographic characteristics of high school Bilibili users. **(A)** Gender distribution: the proportion of females (64.52%) was significantly higher than males (35.48%; χ^2^ = 41.32, *p* < 0.001). **(B)** Grade distribution: grade 10 (42.08%), grade 11 (18.48%), and grade 12 (39.44%), indicating consistently high usage across grade levels. **(C)** Parental education: over 60% of parents held a bachelor's degree (46.53%) or higher (13.53%), suggesting relatively strong family educational backgrounds. **(D)** Residential distribution: core first-tier cities (23.76%) and new first-tier/provincial capitals (29.70%) together accounted for more than half of the sample, highlighting a stronger concentration of users in large cities.

A total of 606 valid questionnaires were collected, yielding a response rate of 99.0%. Gender distribution showed a significantly higher proportion of female students (*n* = 391, 64.52%) than male students (*n* = 215, 35.48%), a difference that was statistically significant (χ^2^ = 41.32, *p* < 0.001), suggesting that female students were more active users of Bilibili (Figure 1A). With respect to grade level, first-year students accounted for the largest proportion (42.08%, *n* = 255), followed by third-year students (39.44%, *n* = 239) and second-year students (18.48%, *n* = 112). Notably, despite the academic pressures faced by third-year students, their participation rate remained close to 40%, indicating that study demands did not entirely suppress entertainment and social needs (Figure 1B).

Regarding socioeconomic status, the majority of respondents came from middle-income households (110,000–300,000 CNY, *n* = 372, 61.39%), while high-income households (≥310,000 CNY) accounted for 21.95% (*n* = 133). This pattern suggests that the overall economic background of Bilibili users falls within the middle-to-upper income range. Parental education levels were relatively high, with nearly half holding a bachelor's degree (46.53%, *n* = 282) and 13.53% (*n* = 82) holding a graduate degree or higher (Figure 1C). In terms of parental occupation, the largest groups were corporate employees (22.77%, *n* = 138), managers or executives (21.95%, *n* = 133), and professionals in science, education, culture, or healthcare (21.78%, *n* = 132), whereas service-sector workers constituted the smallest proportion (3.8%, *n* = 23).

Geographically, 23.76% of respondents were from first-tier cities (Beijing, Shanghai, Guangzhou, and Shenzhen), 29.70% from provincial capitals and “new first-tier” cities, 23.60% from second-tier cities, and 22.94% from third- and fourth-tier cities or towns. These figures indicate that Bilibili maintains broad penetration across urban hierarchies, though with stronger concentration in major metropolitan areas (Figure 1D).

In summary, Bilibili users among high school students are disproportionately female, concentrated in lower grades, and more likely to come from middle- to high-income families with relatively well-educated parents. These demographic patterns suggest stronger platform engagement among female students and those from culturally and economically advantaged households.

### Reliability and validity of the questionnaire

The questionnaire demonstrated high reliability and validity at both the overall and subscale levels. The overall Cronbach's α was 0.931, well above the conventional threshold of 0.80, indicating excellent internal consistency. Subscale Cronbach's α values ranged from 0.81 to 0.90, including entertainment (α = 0.89), social interaction (α = 0.87), cultural identity (α = 0.84), affect (α = 0.90), and behavior (α = 0.88), all meeting or exceeding international standards for reliability.

**Figure 2:**
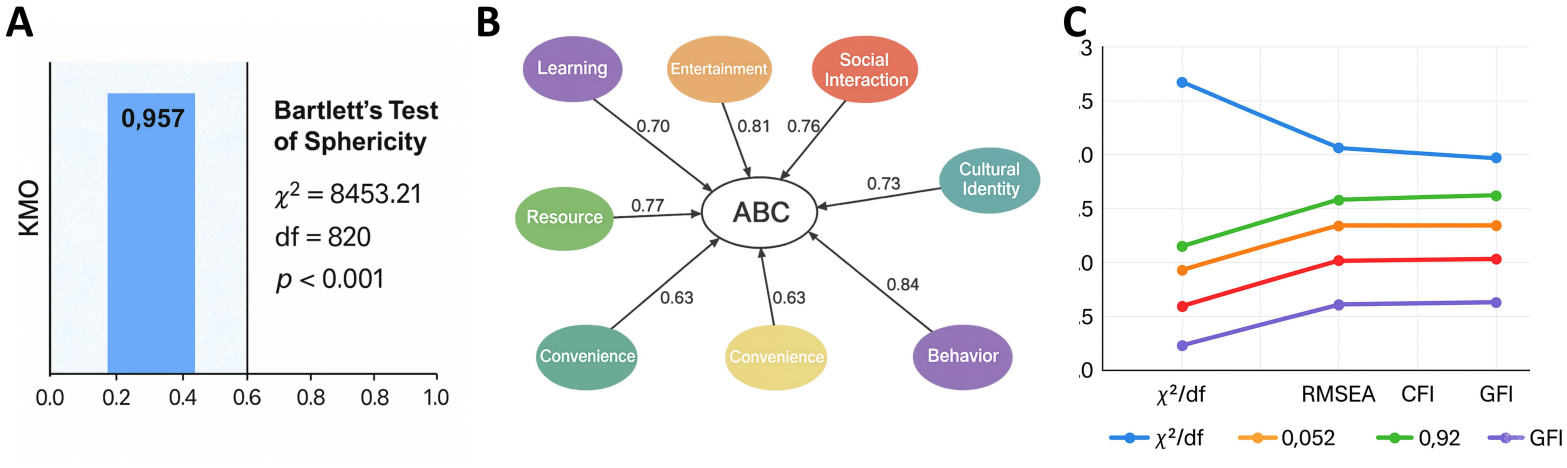
Questionnaire validity and model fit results. **(A)** KMO and Bartlett's tests: KMO = 0.957 and Bartlett's test χ^2^ = 8,453.21, df = 820, *p* < 0.001, confirming suitability for factor analysis. **(B)** CFA path diagram: factor loadings for all eight latent variables—learning, entertainment, social interaction, cultural identity, resources, convenience, affect, and behavior—were above 0.60. **(C)** Model fit indices: χ^2^/df = 2.35, RMSEA = 0.052, CFI = 0.93, TLI = 0.92, and GFI = 0.91, demonstrating good structural validity of the scale.

Validity analysis further supported the robustness of the instrument. The KMO value was 0.957, surpassing the 0.90 benchmark for excellent sampling adequacy. Bartlett's test of sphericity was significant (χ^2^ = 8,453.21, df = 820, *p* < 0.001), confirming strong intercorrelations among variables and suitability for factor analysis. Results of CFA indicated good model fit, *with* χ^2^/df = 2.35 (< 3.0), RMSEA = 0.052 (< 0.08), CFI = 0.93, TLI = 0.92, and GFI = 0.91. Collectively, these findings demonstrate strong structural validity, confirming that the measurement model reliably captured the eight latent dimensions of the ABC model (Figure 2).

In sum, the questionnaire exhibited both high reliability and strong construct validity, providing a sound methodological foundation for subsequent correlation, variance, and path analyses.

### Distribution of multidimensional motivations

Analysis of mean scores across the eight motivational dimensions revealed values ranging from 3.7 to 4.0, suggesting that high school students engage with Bilibili for multiple reasons. Entertainment scored the highest (*M* = 3.96, SD = 0.54), underscoring its central role as the primary motivation, with students frequently using the platform for anime, gaming guides, and variety shows. Convenience followed closely (*M* = 3.95, SD = 0.51), reflecting the platform's user-friendly interface and functional efficiency in sustaining continued use. Affect also scored highly (*M* = 3.92, SD = 0.52), indicating that a sense of belonging and emotional resonance significantly contribute to user stickiness.

**Figure 3:**
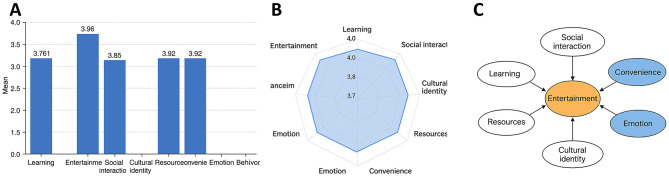
Multidimensional motivations of high school students' Bilibili usage. **(A)** Bar chart of mean scores across eight motivational dimensions, showing entertainment, convenience, and affect scoring higher than learning and cultural identity. **(B)** Radar chart comparing overall performance across dimensions, highlighting the high-value cluster of “entertainment–convenience–affect.” **(C)** Schematic diagram of motivational structure, illustrating a multidimensional framework centered on entertainment, supported by affect and convenience.

Other dimensions, including resources (*M* = 3.89, SD = 0.50) and social interaction (*M* = 3.84, SD = 0.53), were rated positively, highlighting the role of knowledge-rich content and peer interaction in motivating usage. By contrast, learning (*M* = 3.76, SD = 0.49) and cultural identity (*M* = 3.73, SD = 0.47) received comparatively lower scores, suggesting that while educational and cultural content is gaining traction, these drivers remain secondary to entertainment and emotional engagement (Figure 3).

Overall, the findings suggest that high school students' use of Bilibili is shaped by a multidimensional motivational structure characterized by “entertainment at the core, supported by convenience and affect, with learning and cultural identity as supplementary factors.” This distribution highlights the platform's core appeal among adolescents as being primarily entertainment- and emotion-driven.

### Correlation analysis of affect with other dimensions

**Figure 4:**
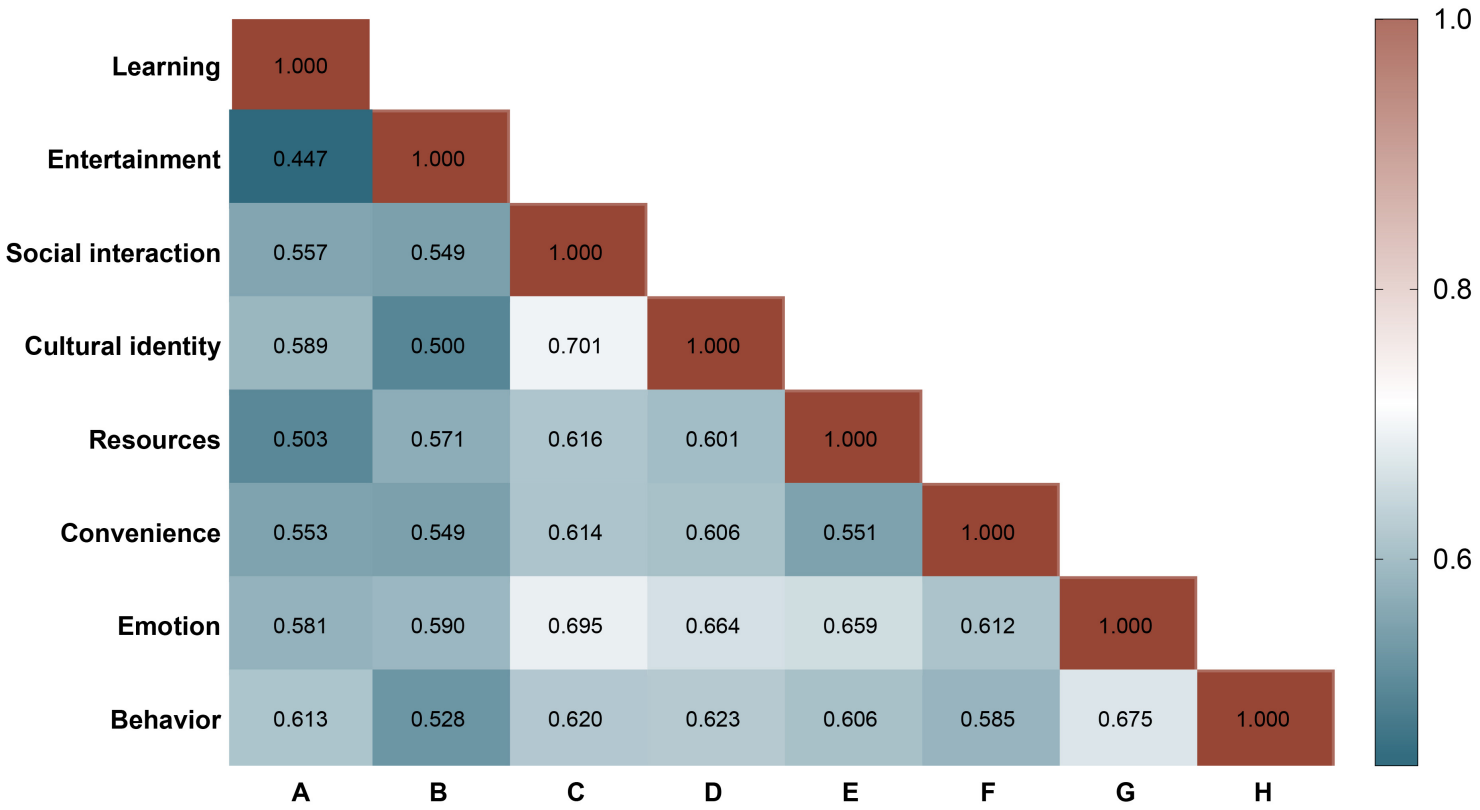
Correlation analysis among the eight study dimensions. The figure presents Pearson's correlation coefficients (*r*) among learning, entertainment, social interaction, cultural identity, resources, convenience, affect, and behavior. All correlations were statistically significant (*p* < 0.01), with the strongest associations observed between social interaction and cultural identity (*r* = 0.701) and between social interaction and affect (*r* = 0.695), underscoring the pivotal role of social interaction in shaping affect and cultural identity.

Pearson correlation analysis (Figure 4) revealed significant positive associations among all eight variables (*p* < 0.01), indicating close interconnections across the dimensions of Bilibili use. The strongest correlation was observed between learning and cultural identity (*r* = 0.589, *p* < 0.001), suggesting that the integration of knowledge-based content with cultural values enhances users' sense of identification and platform engagement. Entertainment was also strongly correlated with affect (*r* = 0.590, *p* < 0.001), highlighting the role of entertainment-oriented content in enriching users' emotional experiences.

**Figure 5:**
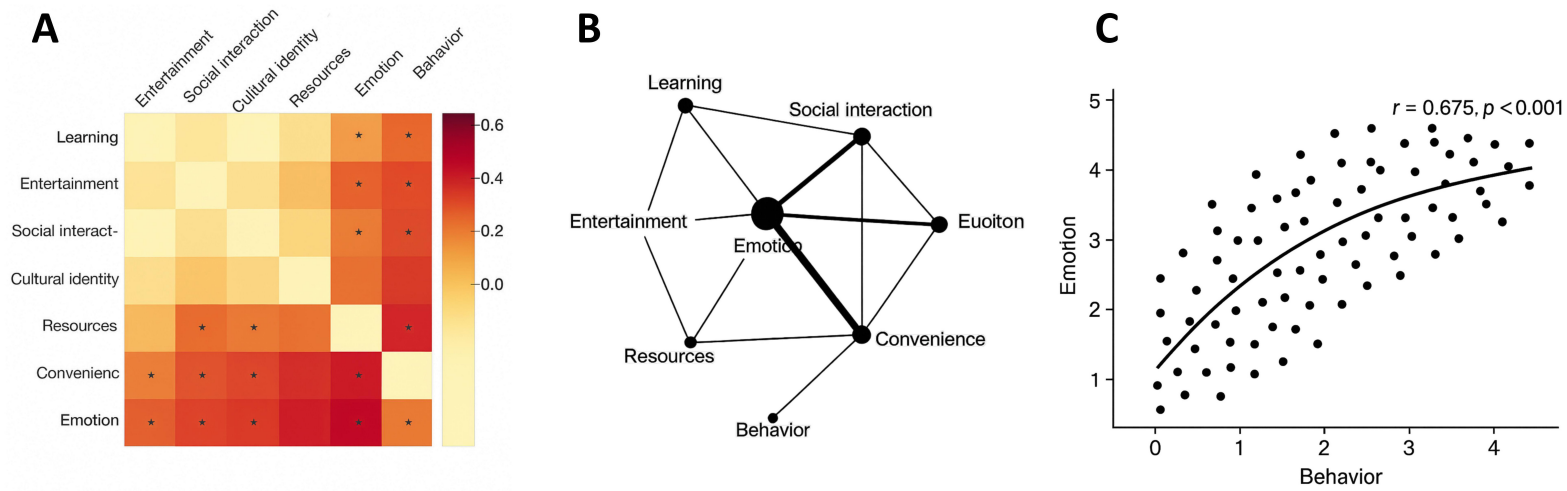
Correlation network of affect and multidimensional variables. **(A)** Heatmap of correlation coefficients among the eight dimensions, where color intensity represents the magnitude of *r* values, with significance indicated at *p* < 0.01. **(B)** Correlation network diagram, where nodes represent variables and edge thickness reflects the strength of correlation, with social interaction, cultural identity, and affect forming the core cluster. **(C)** Scatterplot with fitted curve of affect and behavior (*r* = 0.675, *p* < 0.001), visually demonstrating the strong predictive role of affect in driving user behavior. * indicates statistical significance at *p* < 0.05.

Social interaction showed the strongest associations with both cultural identity (*r* = 0.701, *p* < 0.001) and affect (*r* = 0.695, *p* < 0.001), underscoring its central role in fostering cultural belonging and emotional connection. Moderate-to-strong correlations were also found between affect and resources (*r* = 0.659, *p* < 0.001) as well as convenience (*r* = 0.612, *p* < 0.001), suggesting that content richness and ease of use significantly contribute to emotional engagement. Furthermore, affect was strongly correlated with behavior (*r* = 0.675, *p* < 0.001), indicating that affect not only reflects cognitive outcomes but also directly drives behavioral tendencies (Figure 5). Collectively, these findings support the mediating role of affect in the cognition–behavior relationship, consistent with the theoretical assumptions of the ABC model.

### Comparison by gender and grade

Gender-based subgroup analyses (Tables 3–4) showed no statistically significant differences between male (*n* = 215) and female students (*n* = 391) across the eight dimensions of learning, entertainment, social interaction, cultural identity, resources, convenience, affect, and behavior (all *p* > 0.05). For example, the mean score for entertainment was 3.98 ± 0.53 for females and 3.92 ± 0.56 for males (*t* = 1.12, *p* = 0.26). Similarly, in the affect dimension, females scored 3.94 ± 0.51 compared to 3.89 ± 0.54 for males (*t* = 1.08, *p* = 0.28). These results suggest that gender exerts limited influence on usage behavior, with students demonstrating similar motivational profiles regardless of sex.

**Table 3:** Analysis of differences in eight influencing factors by gender.

**Influencing factor**	**Male (Mean ±SD)**	**Female (Mean ±SD)**	***F***	***P***
Learning	3.7152 ± 0.8	3.7873 ± 0.7	1.187	0.276
Entertainment	4.0125 ± 0.7	3.9294 ± 0.7	1.881	0.171
Social interaction	3.7903 ± 0.8	3.8596 ± 0.7	1.208	0.272
Cultural identity	3.6573 ± 0.9	3.7692 ± 0.8	2.588	0.108
Resources	3.8419 ± 0.7	3.9190 ± 0.6	1.759	0.185
Convenience	3.9045 ± 0.7	3.9811 ± 0.7	1.667	0.197
Emotion	3.8592 ± 0.8	3.9561 ± 0.7	2.466	0.117
Behavior	3.8607 ± 0.8	3.9432 ± 0.7	1.701	0.193

**Table 4:** Analysis of differences in eight influencing factors by age.

**Influencing factor**	**Sophomore (Mean ±SD)**	**Junior** **(Mean ±SD)**	**Senior (Mean ±SD)**	***F***	***p***
Learning	3.6366 ± 0.9	3.8412 ± 0.7	3.7348 ± 0.8	2.939	0.054
Entertainment	4.0270 ± 0.7	4.000 ± 0.7	3.8823 ± 0.7	2.308	0.1
Social interaction	3.9009 ± 0.9	3.8596 ± 0.7	3.7773 ± 0.7	1.299	0.274
Cultural identity	3.6997 ± 0.9	3.7900 ± 0.8	3.6780 ± 0.8	1.241	0.29
Resources	3.8619 ± 0.8	3.8976 ± 0.7	3.8993 ± 0.6	0.13	0.878
Convenience	4.0480 ± 0.8	3.9580 ± 0.6	3.9050 ± 0.7	1.609	0.201
Emotion	3.8619 ± 0.8	3.9291 ± 0.7	3.9217 ± 0.7	0.482	0.618
Behavior	3.8919 ± 0.9	3.9528 ± 0.7	3.8823 ± 0.7	0.611	0.543

**Figure 6:**
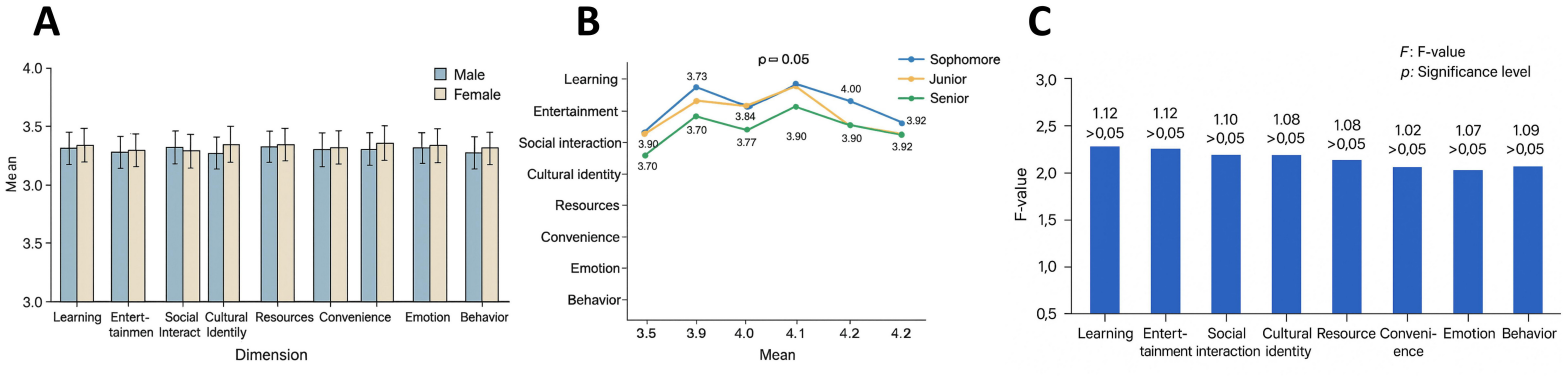
Comparison of gender and grade groups across eight dimensions. **(A)** Bar chart comparing male and female students' mean scores across eight dimensions, showing no significant differences (*p* > 0.05). **(B)** Line chart of mean scores for Grade 10, Grade 11, and Grade 12 students, with similar trends and no significant differences (*p* > 0.05). **(C)** ANOVA results: all *F*-values below 1.2 and significance levels above 0.05, confirming no significant effects of gender or grade on usage motivations.

Grade-level comparisons also revealed no significant differences among first-year (*n* = 255), second-year (*n* = 112), and third-year students (*n* = 239) across the eight dimensions (all *p* > 0.05). For instance, mean scores for learning were 3.77 ± 0.47, 3.73 ± 0.50, and 3.78 ± 0.51 for first-, second, and third-year students, respectively (*F* = 0.32, *p* = 0.72). In social interaction, the respective means were 3.83 ± 0.52, 3.81 ± 0.55, and 3.85 ± 0.53 (*F* = 0.21, *p* = 0.81). These findings indicate that grade level does not significantly shape usage behavior and that students across high school years exhibit a high degree of consistency in their engagement with the platform (Figure 6).

### Impact of school type on social interaction

**Figure 7:**
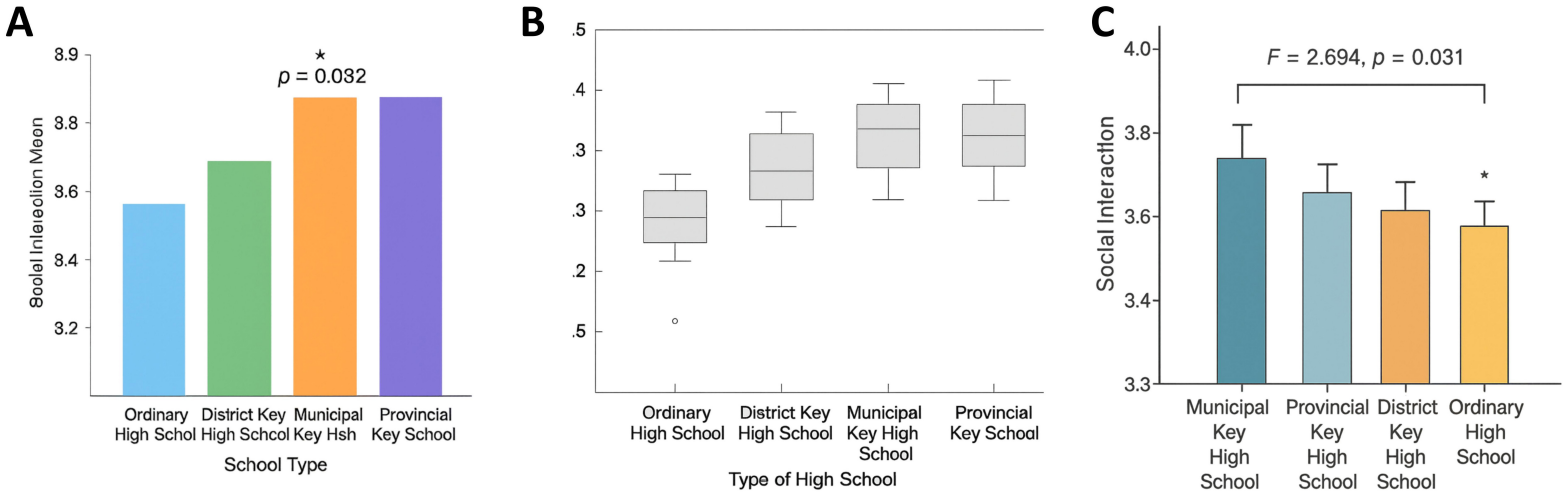
Effect of school type on social interaction. **(A)** Bar chart of mean scores in social interaction among students from regular, district key, municipal key, and provincial key high schools, with municipal key students scoring significantly higher than regular high school students (*p* = 0.032). **(B)** Boxplot showing score distributions and dispersion across the four school types, indicating a generally higher level among municipal key students. **(C)** ANOVA results: *F* = 2.694, *p* = 0.031, with significance observed only between municipal key and regular high schools, while differences among other groups were not significant. * indicates statistical significance at *p* < 0.05.

Group comparisons by school type (Table 5) revealed significant differences in social interaction scores (*F* = 2.694, *p* = 0.031). Students from municipal key high schools reported the highest levels of social interaction (*M* = 3.89, SD = 0.48), significantly exceeding those from regular high schools (*M* = 3.72, SD = 0.45; Bonferroni-adjusted *p* = 0.032). Scores for district key (*M* = 3.83, SD = 0.49) and provincial key high schools (*M* = 3.85, SD = 0.50) were intermediate and not significantly different from municipal key schools (*p* > 0.05). These results suggest that school type influences students' social interaction experiences. In particular, the more resource-rich and socially dynamic environment of municipal key schools may foster greater interaction on Bilibili, whereas students in regular schools may experience relatively weaker engagement, potentially due to heavier academic pressures or peer culture (Figure 7).

**Table 5:** Comparison of social interaction scores among students from different school types.

**Type**	**Provincial key high school (Mean ±SD)**	**Municipal key high school (Mean ±SD)**	**District key high school (Mean ±SD)**	**Ordinary high school (Mean ±SD)**	***F***	***P***
Learning	3.8265 ± 1	3.7869 ± 0.8	3.7400 ± 0.6	3.6632 ± 0.7	0.878	0.452
Entertainment	4.0306 ± 0.8	3.9373 ± 0.7	3.9489 ± 0.6	3.9588 ± 0.6	0.42	0.739
Social interaction	3.8912 ± 0.9	3.9020 ± 0.7	3.7911 ± 0.7	3.6701 ± 0.7	2.694	0.045
Cultural identity	3.7415 ± 1	3.7948 ± 0.8	3.6933 ± 0.7	3.6014 ± 0.8	1.449	0.228
Resources	3.9252 ± 0.9	3.9176 ± 0.7	3.9289 ± 0.6	3.7320 ± 0.7	2.138	0.094
Convenience	4.1054 ± 0.8	3.9556 ± 0.7	3.8911 ± 0.6	3.8935 ± 0.7	2.22	0.085
Emotion	3.9830 ± 0.9	3.9542 ± 0.7	3.8889 ± 0.6	3.8247 ± 0.7	1.088	0.353
Behavior	3.9422 ± 0.9	3.9569 ± 0.7	3.9156 ± 0.6	3.7698 ± 0.8	1.56	0.198

The table reports the mean social interaction scores (M ± SD) of students from regular high schools (*n* = 99), district key high schools (*n* = 151), municipal key high schools (*n* = 256), and provincial key high schools (*n* = 100). One-way ANOVA revealed a significant overall difference (*F* = 2.694, *p* = 0.031), and *post hoc* tests indicated that students from municipal key high schools scored significantly higher in social interaction than those from regular high schools.

### Impact of parental occupation on cultural identity and behavior

**Figure 8:**
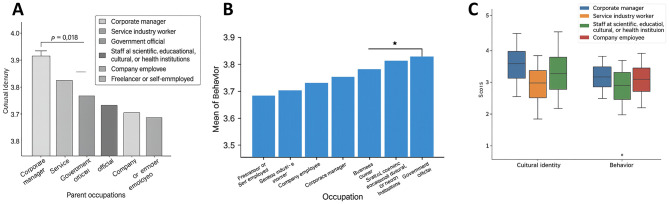
Effect of parental occupation on cultural identity and behavior. **(A)** Bar chart of mean scores in cultural identity, showing children of enterprise managers scoring significantly higher than those of service industry workers (*p* = 0.018). **(B)** Bar chart of mean scores in behavior, with children of professionals in education, science, culture, and healthcare scoring significantly higher than children of company staff (*p* = 0.041). **(C)** Boxplot illustrating the distribution and variation across four parental occupational groups, further highlighting the potential influence of occupational background on students' cultural identity and behavioral tendencies. * indicates statistical significance at *p* < 0.05.

Parental occupation was significantly associated with cultural identity and behavior (Figure 8). ANOVA results showed significant group differences for cultural identity (*F* = 3.121, *p* = 0.021) and behavior (*F* = 2.946, *p* = 0.034). Specifically, children of corporate executives scored highest on cultural identity (*M* = 3.84, SD = 0.46), significantly higher than those of service-sector workers (*M* = 3.61, SD = 0.49; Bonferroni-adjusted *p* = 0.018). For behavior, students with parents employed in science, education, culture, or healthcare scored significantly higher (*M* = 3.88, SD = 0.44) than those whose parents were corporate employees (*M* = 3.73, SD = 0.47; *p* = 0.041).

These findings suggest that family occupational background contributes to shaping adolescents' cultural identification and behavioral engagement. Students from families with greater cultural capital or social resources may benefit from parental educational values, cultural participation, and indirect support of their social environment, thereby reporting stronger identity and higher levels of platform engagement.

### Impact of family income on entertainment, social interaction, and cultural identity

**Figure 9:**
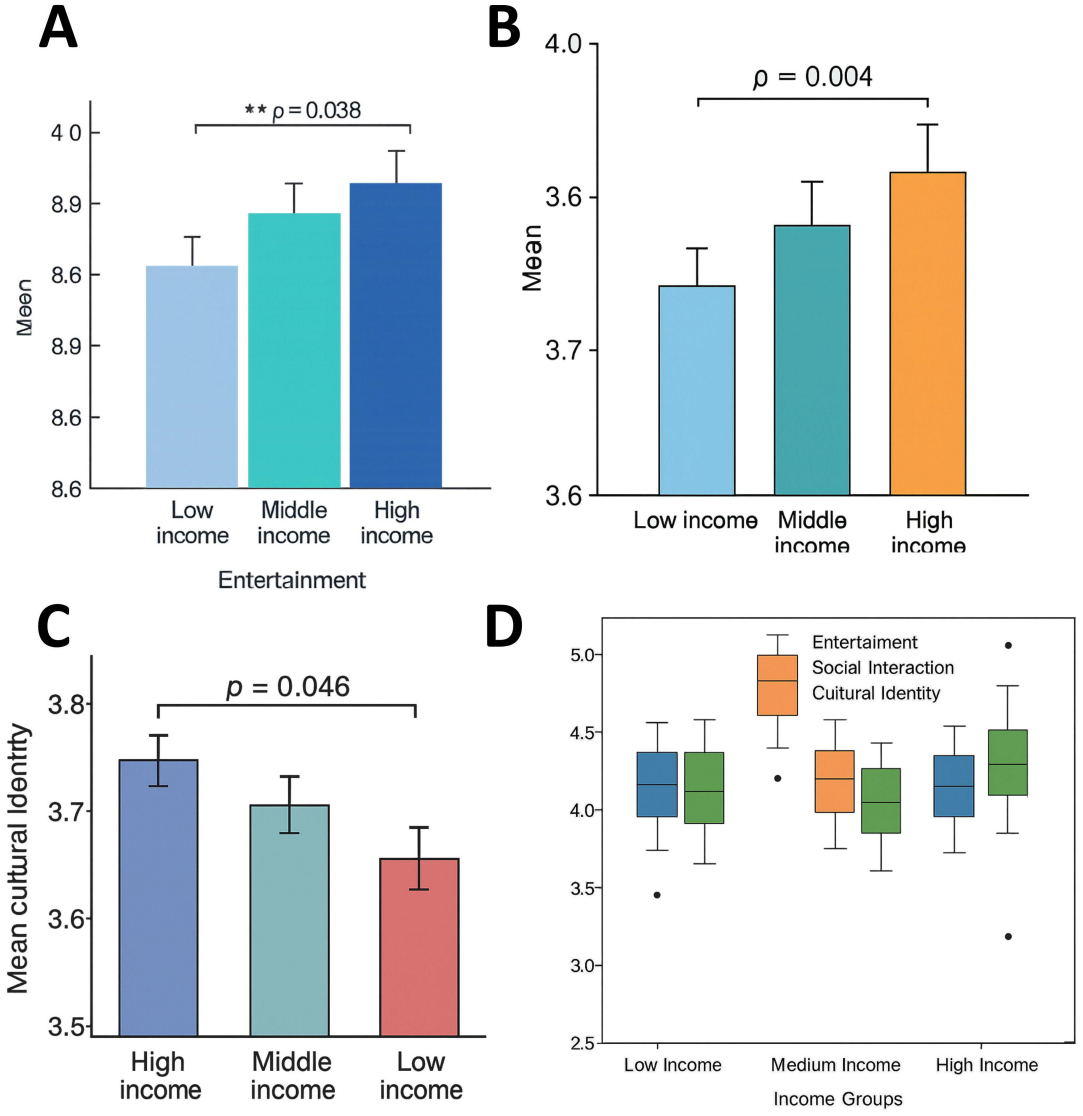
Effect of family income on entertainment, social interaction, and cultural identity. **(A)** Bar chart of mean scores for entertainment across three income groups, with high-income students scoring significantly higher than low-income students (*p* = 0.038). **(B)** Bar chart of mean scores for social interaction, showing high-income students significantly outperforming low-income students (*p* = 0.004), with middle-income students in between. **(C)** Bar chart of mean scores for cultural identity, with high-income students scoring significantly higher than low-income students (*p* = 0.046). **(D)** Boxplot comparing distributions across the three income groups, further illustrating the positive influence of economic conditions on entertainment, social interaction, and cultural identity. ** indicates statistical significance at *p* < 0.01.

Significant group differences were also observed across family income levels (Figure 9). ANOVA results indicated significant effects on entertainment (*F* = 2.766, *p* = 0.041), social interaction (*F* = 3.767, *p* = 0.005), and cultural identity (*F* = 2.533, *p* = 0.048). Students from high-income households (≥310,000 CNY, *n* = 133) scored highest in social interaction (*M* = 3.92, SD = 0.47), significantly exceeding those from low-income households ( ≤ 100,000 CNY, *n* = 101; *M* = 3.68, SD = 0.50; Bonferroni-adjusted *p* = 0.004). Students from middle-income households (110,000–300,000 CNY, *n* = 372) scored in between (*M* = 3.83, SD = 0.48).

In entertainment, both the high-income (*M* = 4.01, SD = 0.52) and middle-income groups (*M* = 3.95, SD = 0.53) scored higher than the low-income group (*M* = 3.82, SD = 0.56), with the high-vs low-income difference reaching significance (*p* = 0.038). For cultural identity, the high-income group (*M* = 3.81, SD = 0.44) also scored significantly higher than the low-income group (*M* = 3.66, SD = 0.48; *p* = 0.046).

These results indicate that economic conditions shape adolescents' entertainment preferences, social activity, and cultural identification. Students from more advantaged households are more likely to participate in social interaction and cultural content consumption on Bilibili, whereas those from less affluent backgrounds show comparatively weaker engagement in these dimensions.

### Limited effect of residential location

**Figure 10:**
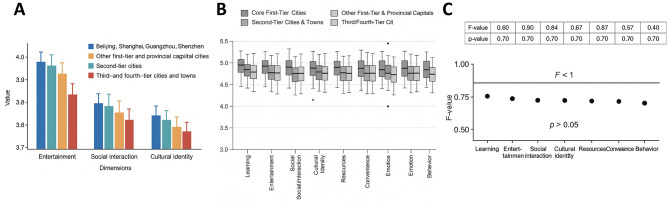
Influence of residential location on Bilibili usage behavior among high school students. **(A)** Bar chart of mean scores in entertainment, social interaction, and cultural identity across four residential tiers, with no significant differences observed (all *p* > 0.05). **(B)** Boxplot displaying the overall distribution across all eight dimensions, showing high overlap and no significant differences. **(C)** ANOVA results: all *F*-values below 1.0 and all *p*-values above 0.05, further confirming that residential location had no significant effect on usage behavior.

Comparisons across residential tiers—core first-tier cities (Beijing, Shanghai, Guangzhou, Shenzhen; *n* = 144), other first-tier and provincial capital cities (*n* = 180), second-tier cities (*n* = 143), and third-/fourth-tier cities and towns (*n* = 139)—showed no statistically significant differences across any of the eight dimensions (all *p* > 0.05; Figure 10). For example, in the entertainment dimension, mean scores were 3.97 ± 0.52 for core first-tier cities, 3.94 ± 0.55 for provincial capitals/new first-tier cities, 3.91 ± 0.51 for second-tier cities, and 3.89 ± 0.56 for smaller cities and towns, with no significant differences (*F* = 0.42, *p* = 0.74).

A similar pattern was observed in social interaction: 3.86 ± 0.48 for core first-tier cities, 3.84 ± 0.50 for provincial capitals/new first-tier cities, 3.82 ± 0.52 for second-tier cities, and 3.81 ± 0.51 for smaller cities, again without significant variation (*F* = 0.39, *p* = 0.76). No significant differences emerged in cultural identity, learning, resources, convenience, affect, or behavior. These findings suggest that place of residence is not a key determinant of high school students' Bilibili use, reflecting relatively balanced penetration and user experiences across urban hierarchies. This also indicates that Bilibili's content and functional design exhibit strong universality across regions.

### Path analysis and the mediating role of affect

**Figure 11:**
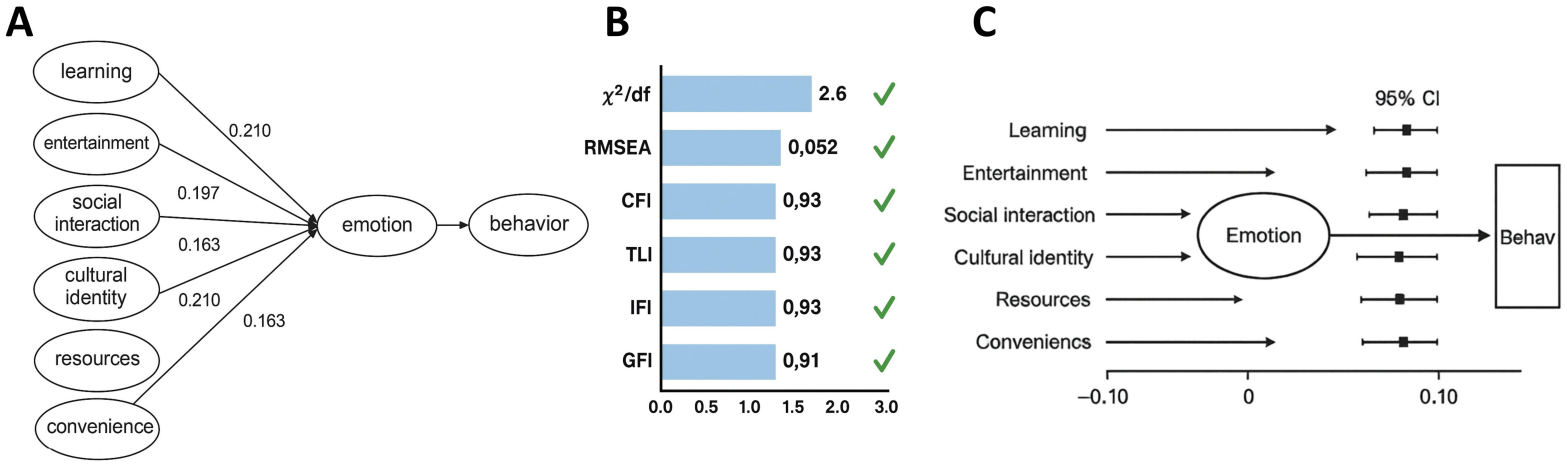
Structural model of path analysis and the mediating role of affect. **(A)** SEM path diagram illustrating the positive effects of six cognitive dimensions—learning, entertainment, social interaction, cultural identity, resources, and convenience—on affect, and the central role of affect in predicting behavior. Significant paths are shown as solid lines with coefficients indicated. **(B)** Model fit statistics demonstrating acceptable indices: χ^2^/df, RMSEA, CFI, TLI, IFI, and GFI all within ideal thresholds. **(C)** Bootstrap mediation test results, with all 95% confidence intervals for indirect effects excluding zero, confirm the significant mediating role of affect.

The SEM constructed in AMOS demonstrated good fit (χ^2^/df = 2.62, RMSEA = 0.052, CFI = 0.93, TLI = 0.92, IFI = 0.93, GFI = 0.91), meeting international standards and indicating strong alignment between the theoretical model and empirical data (Figure 11). Path analysis revealed that all six cognitive dimensions positively influenced affect, with the strongest effects from entertainment (β = 0.210, *p* < 0.01) and resources (β = 0.210, *p* < 0.01). Social interaction (β = 0.197, *p* < 0.01), cultural identity (β = 0.163, *p* < 0.05), learning (β = 0.113, *p* < 0.05), and convenience (β = 0.118, *p* < 0.05) also showed significant positive effects.

Affect exerted the most substantial direct effect on behavior (β = 0.939, *p* < 0.001), underscoring its central role in the model. Bootstrap mediation tests (5,000 resamples) further confirmed that all cognitive dimensions exerted significant indirect effects on behavior through affect, with 95% confidence intervals not crossing zero. These results demonstrate that affect serves as a complete or partial mediator in the cognition–behavior relationship, functioning not merely as an outcome of cognition but as a pivotal bridge driving user behavior.

## Discussion

This study, based on data from 606 high school students, validated the cognition-affect-behavior pathway through which multiple cognitive factors affect behavior via affect. All six cognitive dimensions—entertainment, learning, social interaction, cultural identity, resources, and convenience—significantly predicted affect, and affect exerted the strongest influence on behavior (β = 0.939, *p* < 0.05), consistent with studies highlighting the central role of affect in user decision-making (Plackett et al., 2023; McHugh et al., 2024). Entertainment was the primary motivation (*M* = 3.9589) and showed a strong association with affect (*r* = 0.590), reflecting an “affective amplification effect” (Martin and MacDonald, 2020). Although learning motivation was secondary (*M* = 3.7617), it still positively predicted affect (β ≈ 0.15), aligning with prior findings on its complementary role (Lacey et al., 2024; Maloney et al., 2013). Social interaction and cultural identity were highly coupled (*r* = 0.701) and jointly enhanced affect, while resource richness and convenience also contributed to affective identification. Family income, school type, and parental occupation significantly influenced entertainment, social interaction, and cultural identity (*F* = 2.766–3.767), reflecting the multi-path effects of social capital on affect and behavior (Ma et al., 2023). Overall, adolescents on Bilibili exhibited a hybrid motivational structure that integrates entertainment, learning, and cultural belonging, highlighting the importance of platform-specific cultural features and the need for cross-cultural comparison.

Compared with existing research on Bilibili and short-video platforms, this study represents substantial progress in theoretical framework, target population, variable structure, mechanism testing, and methodological rigor. It is the first to systematically apply the ABC model to the context of high school students' use of Bilibili, constructing a complete ABC pathway that advances beyond prior work focused mainly on platform operations or technological affordances (Di Spirito et al., 2023). By targeting high school students—rather than general “youth”—the study captures their distinctive learning needs, entertainment reliance, and sensitivity to cultural identity (Murthy et al., 2023). At the variable level, incorporating six cognitive dimensions extends beyond single-motivation perspectives and reveals the synergistic structure between social interaction and cultural identity (*r* = 0.701). Mechanistically, the study rigorously validated the mediating role of affect (β = 0.939), shifting from “listing factors” to “explaining mechanisms” (Jafari et al., 2024). Methodologically, the use of structural equation modeling, multilevel variance analysis, and Bootstrap mediation tests provides a more robust evidence chain than prior qualitative or correlational studies (Kim et al., 2024). Collectively, the study demonstrates substantial improvement in theoretical depth, variable integration, mechanism clarification, and methodological rigor.

Within the broader context of social inequality, this study reveals the unique role of affective mechanisms from the perspective of digital inequality. Existing research largely focuses on disparities in access, skills, and usage, while overlooking the emotional mechanisms underlying these differences (Soomro et al., 2020). Although studies drawing on Bourdieu's framework discuss cultural capital and “digital habitus,” they have seldom addressed the mediating role of affect in capital conversion (Ragnedda and Ruiu, 2017). This study shows that socioeconomic capital—such as family income and school type—not only shapes how students engage with different cognitive dimensions on the platform but also alters behavioral tendencies through affect. This suggests that disparities in real-world capital are amplified by affect within digital environments. The mediating role of affect in the ABC model can thus be seen as a mechanism of digital reproduction, illustrating how socioeconomic inequality permeates online behavior through individualized emotional experiences. This highlights the need to address affective barriers among disadvantaged groups to prevent digital inequality from persisting at affective and behavioral levels.

Within Bourdieu's theoretical framework, the findings further indicate that affect serves as a key mechanism through which digital cultural capital is transformed into platform behavior. Prior research has shown that digital cultural and social capital shape learners' online practices (Feng and Tan, 2024), school-related internet use exhibits clear stratification (Jin et al., 2019), and digital inequality remains embedded within broader social structures (Kuhn et al., 2023). Online behavior studies further reveal that users with lower social status are more likely to be confined to entertainment-oriented information spaces (Xu et al., 2020), and that affective experiences are tightly linked to class-based habitus (Leaney, 2018). Consistent with these findings, the present results show that cognitive evaluations of Bilibili influence behavior primarily through affect (β = 0.939, *p* < 0.001), and students from high-income families, key schools, and higher occupational backgrounds score higher on affect-related dimensions. This indicates that positive affective feedback forms a stratified pathway through which digital cultural resources are converted into differentiated behavioral engagement, reinforcing social inequality within digital settings.

From a demographic perspective, the study presents clear stratification patterns that contextualize the social embeddedness of affective mediation. Although the sample overall comes from families with relatively abundant cultural and economic capital, students from high-income families, key schools, and higher occupational backgrounds consistently scored higher on entertainment, social interaction, and cultural identity, dimensions closely tied to affect generation, making them more likely to enter the positive cognition–affect-behavior pathway. The strong impact of affect on behavior (β = 0.939) further indicates that this mediating mechanism is not equally accessible but favors resource-advantaged groups. This aligns with findings that parental socioeconomic status shapes adolescents' digital use and emotional experiences (Männikkö et al., 2020; Becker, 2000), that demographic differences exist in information technology orientations (Koivusilta et al., 2007), and that links between digital use and well-being show structural inequalities across SES groups (Bohnert and Gracia, 2023). Therefore, the affective mediation mechanism can be understood as a form of digital cultural capital conversion embedded in social structure: resource-rich groups are better positioned to transform digital opportunities into positive affect and sustained participation, while disadvantaged groups are more likely to experience marginalization. Thus, demographic structure should be considered essential when interpreting the findings and their broader applicability.

The findings show that affect's mediating role in the ABC model varies by socioeconomic background, meaning disadvantaged students have fewer opportunities to generate positive emotional experiences that activate the cognition-affect-behavior pathway. Practically, schools can strengthen emotional support through peer assistance, positive feedback, and guided participation in learning or interest communities to enhance disadvantaged students' emotional wellbeing. Platforms can increase the visibility of educational and community-supportive content and adopt emotion-friendly interaction designs to make positive affect more accessible. Such coordinated interventions help ensure that disadvantaged students can also benefit from affective pathways and reduce the reproduction of digital inequality.

This study has several limitations that provide directions for future research. First, the cross-sectional design limits causal inference. Longitudinal tracking, for example, following the same cohort throughout 3 years of high school, would help examine dynamic changes in cognition-affect-behavior relationships and assess the temporal stability of affective mediation. Experimental manipulation, such as A/B testing interface elements related to convenience or resource presentation, could further clarify the causal effects of specific cognitive dimensions. Second, the sample was drawn from a limited region, which may affect generalizability. Cross-cultural comparisons applying the model to adolescent users of Bilibili, YouTube, and TikTok could illuminate how platform culture and algorithmic mechanisms moderate the ABC pathway. Nationally stratified sampling covering urban, suburban, and rural areas would also enhance representativeness. Third, reliance on self-reported data may introduce bias. Future research should incorporate behavioral logs (e.g., content preferences, bullet-screen/comment frequency, usage duration) for triangulation, and employ multimodal physiological measures such as eye tracking or skin conductance to capture difficult-to-report affective reactions and more precisely validate affect's mediating role.

**Figure 12:**
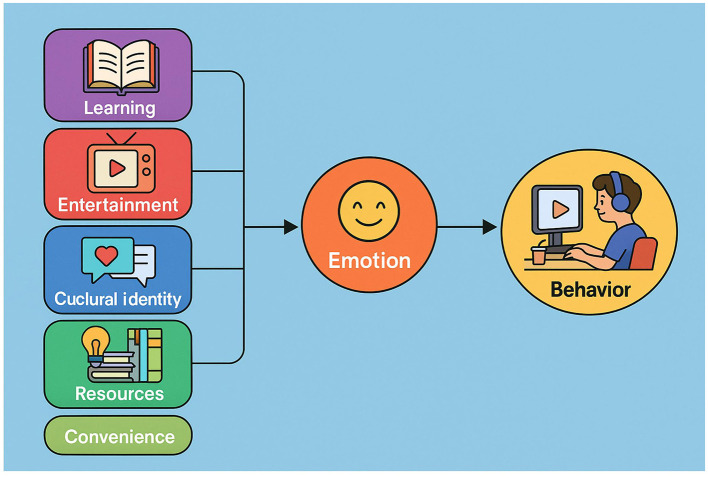
Mechanism of high school students' Bilibili usage behavior based on the ABC model.

In conclusion, this study systematically investigated the cognition-affect-behavior pathways, revealing the central role of affect in shaping high school students' engagement with Bilibili. The findings enrich the application of the ABC model to adolescent populations and provide empirical support for optimizing platform design and enhancing user engagement from an affective perspective. Scientifically, the study advances understanding of the psychological and social mechanisms underlying adolescents' digital practices; practically, it offers affect-informed guidance for short-video and educational platform design. Despite its limitations, the study lays a strong foundation for understanding affect-driven digital behaviors among adolescents and points to promising directions for future interdisciplinary and cross-cultural research (Figure 12).

## Data Availability

The original contributions presented in the study are included in the article/Supplementary material, further inquiries can be directed to the corresponding author.
