# Supplementary material for: Socioeconomic stratification in adolescent digital engagement: cultural capital, emotional mediation, and bilibili usage patterns in Chinese high schools
Source: Front Sociol. 2026 Jan 13;10:1696513. doi: 10.3389/fsoc.2025.1696513 (PMC12834749; doi:10.3389/fsoc.2025.1696513)
Supplement: Supplementary file 1 [file Supplementary_file_1.docx]

Dear Participant,

Thank you for taking the time out of your busy schedule to participate in this survey! The purpose of this questionnaire is to understand your usage of Bilibili. The data collected will be used solely for research purposes. Your answers will be kept strictly confidential, and the survey is anonymous. Please select the options that best reflect your usual experience. It should take about 5 minutes to complete this questionnaire. Thank you very much for your support! Wishing you a pleasant day ahead!

1. What is your gender?

A. Male

B. Female

2. What grade are you in?

A. Grade 10 (Junior 1)

B. Grade 11 (Junior 2)

C. Grade 12 (Senior 1)

3. What is your academic ranking within your grade?

A. Top 5%

B. 6% to 25%

C. 26% to 50%

D. 51% to 75%

E. Below 76%

4. What type of high school do you attend?

A. Provincial Key High School (e.g., Shanghai's top four schools)

B. Municipal Key High School

C. District Key High School

D. Ordinary High School

5. What is the highest level of education attained by either of your parents?

A. High School or below

B. Associate Degree

C. Bachelor's Degree

D. Graduate Degree or above

6. What is the occupation of either of your parents?

A. Government Official

B. Staff Member of Scientific, Educational, Cultural, or Health Institutions

C. Business Owner

D. Corporate Manager

E. Corporate Employee

F. Service Industry Worker

G. Freelancer

7. What is your family's annual income?

A. Less than 100,000 RMB

B. 110,000 to 200,000 RMB

C. 210,000 to 300,000 RMB

D. 310,000 to 400,000 RMB

E. 410,000 to 500,000 RMB

8. Where are you from?

A. Beijing, Shanghai, Guangzhou, Shenzhen

B. Other first-tier cities and provincial capitals outside of Beijing, Shanghai, Guangzhou, Shenzhen

C. Second-tier cities

D. Third and fourth-tier cities and towns

Please answer the following questions truthfully based on your usual experiences. Choose from "Strongly Disagree", "Disagree", "Neutral", "Agree", "Strongly Agree".

9. Compared to other software, I prefer studying on Bilibili.

A. Strongly Disagree

B. Disagree

C. Neutral

D. Agree

E. Strongly Agree

10. Compared to other software, I receive more tutorial-related videos on Bilibili.

A. Strongly Disagree

B. Disagree

C. Neutral

D. Agree

E. Strongly Agree

11. Compared to other software, I achieve the highest learning efficiency on Bilibili.

A. Strongly Disagree

B. Disagree

C. Neutral

D. Agree

E. Strongly Agree

12. Compared to other software, I get more relaxation on Bilibili.

A. Strongly Disagree

B. Disagree

C. Neutral

D. Agree

E. Strongly Agree

13. Compared to other software, I obtain more entertainment information on Bilibili.

A. Strongly Disagree

B. Disagree

C. Neutral

D. Agree

E. Strongly Agree

14. Compared to other software, I see more entertainment programs on Bilibili.

A. Strongly Disagree

B. Disagree

C. Neutral

D. Agree

E. Strongly Agree

15. Compared to other software, I prefer the community on Bilibili.

A. Strongly Disagree

B. Disagree

C. Neutral

D. Agree

E. Strongly Agree

16. Compared to other software, I have more topics to discuss with close friends on Bilibili.

A. Strongly Disagree

B. Disagree

C. Neutral

D. Agree

E. Strongly Agree

17. Compared to other software, if my friends use Bilibili, I also use Bilibili.

A. Strongly Disagree

B. Disagree

C. Neutral

D. Agree

E. Strongly Agree

18. Compared to other software, I prefer the bullet comments on Bilibili.

A. Strongly Disagree

B. Disagree

C. Neutral

D. Agree

E. Strongly Agree

19. Compared to other software, I prefer communicating with my peers on Bilibili.

A. Strongly Disagree

B. Disagree

C. Neutral

D. Agree

E. Strongly Agree

20. Compared to other software, I prefer uploading content on Bilibili.

A. Strongly Disagree

B. Disagree

C. Neutral

D. Agree

E. Strongly Agree

21. Compared to other software, I prefer the exclusive content on Bilibili.

A. Strongly Disagree

B. Disagree

C. Neutral

D. Agree

E. Strongly Agree

22. Compared to other software, I prefer watching re-uploaded videos on Bilibili.

A. Strongly Disagree

B. Disagree

C. Neutral

D. Agree

E. Strongly Agree

23. Compared to other software, I prefer watching original uploads on Bilibili.

A. Strongly Disagree

B. Disagree

C. Neutral

D. Agree

E. Strongly Agree

24. Compared to other software, Bilibili does not display ads before or after videos.

A. Strongly Disagree

B. Disagree

C. Neutral

D. Agree

E. Strongly Agree

25. Compared to other software, I find Bilibili more convenient to use.

A. Strongly Disagree

B. Disagree

C. Neutral

D. Agree

E. Strongly Agree

26. Compared to other software, I find the search function on Bilibili more efficient.

A. Strongly Disagree

B. Disagree

C. Neutral

D. Agree

E. Strongly Agree

27. In general, compared to other software, I prefer Bilibili.

A. Strongly Disagree

B. Disagree

C. Neutral

D. Agree

E. Strongly Agree

28. In general, compared to other software, I believe Bilibili better represents the culture of the younger generation.

A. Strongly Disagree

B. Disagree

C. Neutral

D. Agree

E. Strongly Agree

29. In general, compared to other software, I believe Bilibili is better at guiding youth towards the right path.

A. Strongly Disagree

B. Disagree

C. Neutral

D. Agree

E. Strongly Agree

30. My frequency of using Bilibili is similar to that of my classmates.

A. Strongly Disagree

B. Disagree

C. Neutral

D. Agree

E. Strongly Agree

31. I am very willing to recommend Bilibili to my classmates.

A. Strongly Disagree

B. Disagree

C. Neutral

D. Agree

E. Strongly Agree

32. I will continue to use Bilibili.

A. Strongly Disagree

B. Disagree

C. Neutral

D. Agree

E. Strongly Agree
