# Supplementary figures and images for: Socioeconomic stratification in adolescent digital engagement: cultural capital, emotional mediation, and bilibili usage patterns in Chinese high schools
Source: Front Sociol. 2026 Jan 13;10:1696513. doi: 10.3389/fsoc.2025.1696513 (PMC12834749; doi:10.3389/fsoc.2025.1696513)

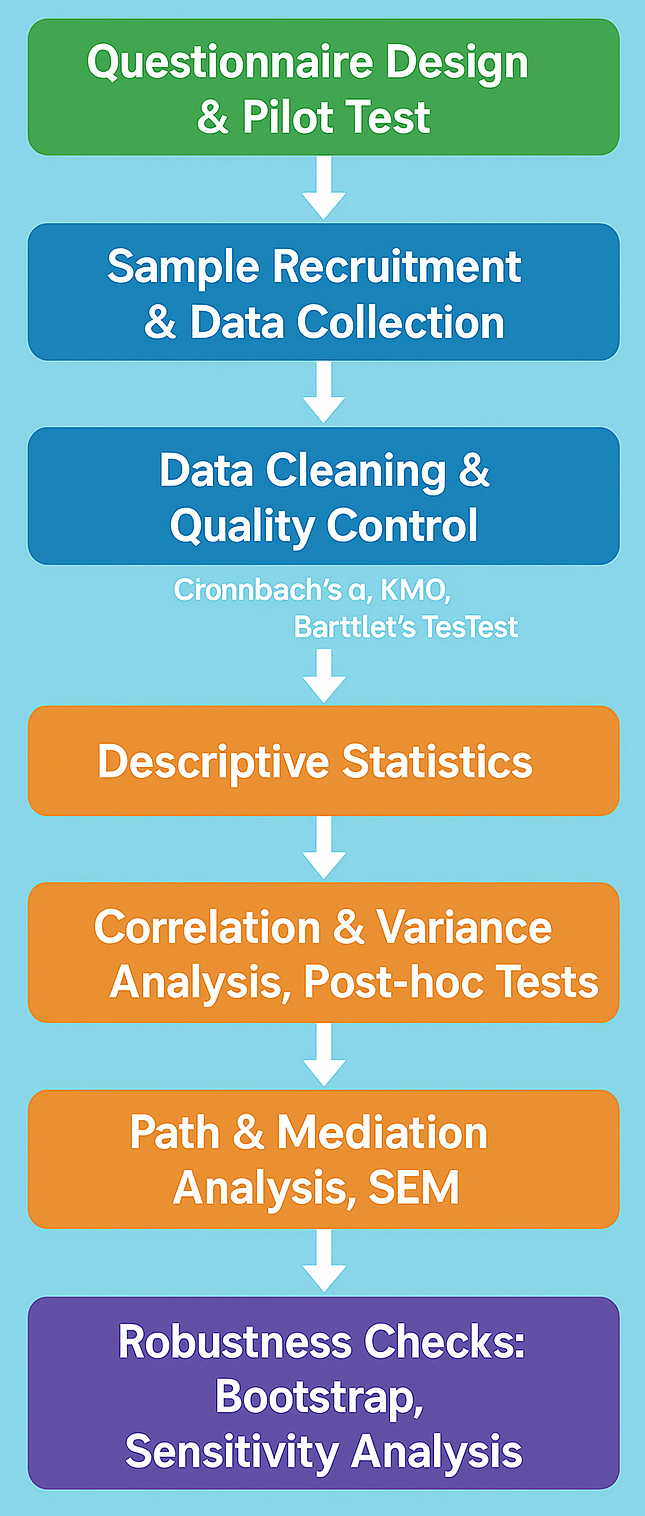

Supplement: Supplementary file 2 [file Image_1.png]

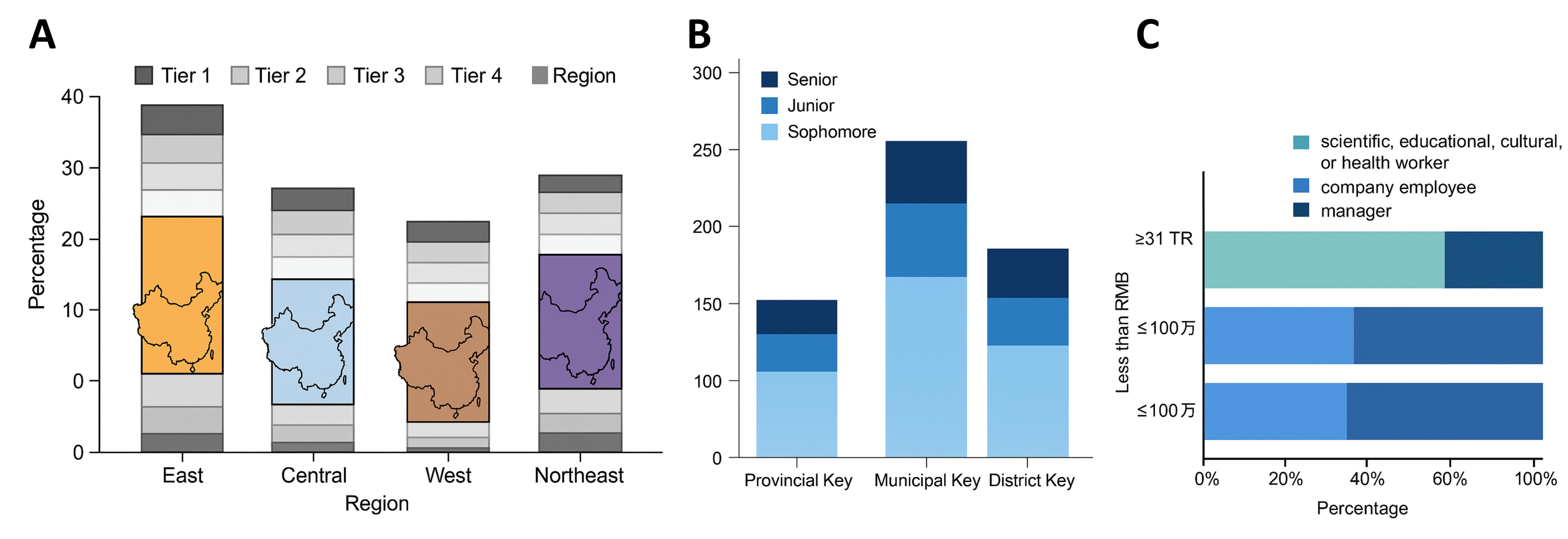

Supplement: Supplementary file 3 [file Image_2.jpg]

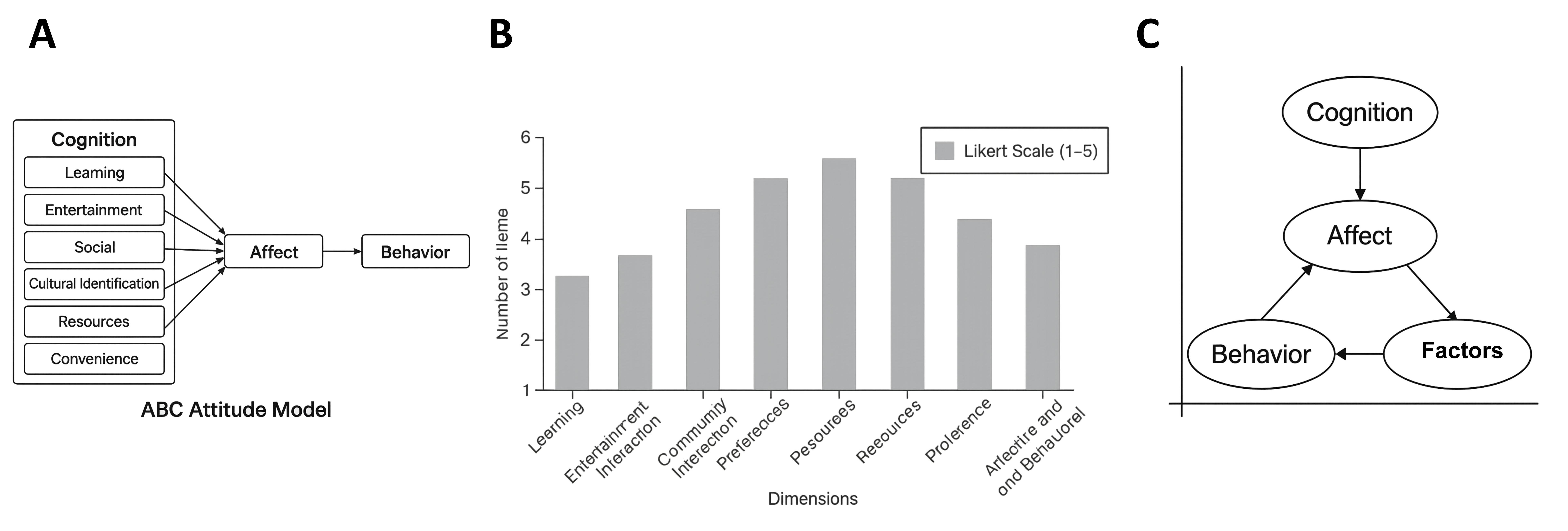

Supplement: Supplementary file 4 [file Image_3.jpg]
